# Is there a linear relationship between the Brief Psychiatric Rating Scale and the Clinical Global Impression-Schizophrenia scale? A retrospective analysis

**DOI:** 10.1186/1471-244X-10-105

**Published:** 2010-12-07

**Authors:** Jitsuki Sawamura, Shigeru Morishita, Jun Ishigooka

**Affiliations:** 1 Department of Psychiatry, Tokyo Women's Medical University, Tokyo, Japan; 2 Depression Prevention Medical Center, Kyoto Jujo Rehabilitation Hospital, Kyoto, Japan

## Abstract

**Background:**

Although the Brief Psychiatric Rating Scale (BPRS) is widely used for evaluating patients with schizophrenia, it has limited value in estimating the clinical weight of individual symptoms. The aim of this study was 4-fold: 1) to investigate the relationship of the BPRS to the Clinical Global Impression-Schizophrenia Scale (CGI-SCH), 2) to express this relationship in mathematical form, 3) to seek significant symptoms, and 4) to consider a possible modified BPRS subscale.

**Methods:**

We evaluated 150 schizophrenia patients using the BPRS and the CGI-SCH, then examined the scatter plot distribution of the two scales and expressed it in a mathematical equation. Next, backward stepwise regression was performed to select BPRS items that were highly associated with the CGI-SCH. Multivariate regression was conducted to allocate marks to individual items, proportional to their respective magnitude. We assessed the influence of modifications to the BPRS in terms of Pearson's r correlation coefficient and r-squared to evaluate the relationship between the two scales. Utilizing symptom weighting, we assumed a possible BPRS subscale.

**Results:**

By plotting the scores for the two scales, a logarithmic curve was obtained. By performing a logarithmic transformation of the BPRS total score, the curve was modified to a linear distribution, described by [CGI-SCH] = 7.1497 × log_10_[18-item BPRS] - 6.7705 (p < 0.001). Pearson's r for the relationship between the scales was 0.7926 and r-squared was 0.7560 (both p < 0.001). Applying backward stepwise regression using small sets of items, eight symptoms were positively correlated with the CGI-SCH (p < 0.005) and the subset gave Pearson's r of 0.8185 and r-squared of 0.7198. Further selection at the multivariate regression yielded Pearson's r of 0.8315 and r-squared of 0.7036. Then, modification of point allocation provided Pearson's r of 0.8339 and r-squared of 0.7036 (all these p < 0.001). A possible modified BPRS subscale, "the modified seven-item BPRS", was designed.

**Conclusions:**

Limited within our data, a logarithmic relationship was assumed between the two scales, and not only individual items of the BPRS but also their weightings were considered important for a linear relationship and improvement of the BPRS for evaluating schizophrenia.

## Background

Schizophrenia is a serious mental disorder characterized by a number of symptoms. To evaluate the effects of treatment for schizophrenia, it is important to assign quantitative values to the symptoms. Many rating scales have been used to evaluate various symptomatic domains in schizophrenia [1]. This has led to confusion regarding the suitability of the different scales available, not only in relation to evaluation and treatment of the disease but also in research and clinical studies of the effects of medication. Currently, consensus is lacking about which rating scales are appropriate to evaluate schizophrenia. Evaluation scales that are relevant, quick, user-friendly, graduated at equal intervals and with high linearity are needed to facilitate measurement-based treatment of schizophrenia. The Brief Psychiatric Rating Scale [2] is one of the standard instruments used most frequently in daily practice for evaluating the severity of schizophrenia. Also popular are the Clinical Global Impression-Schizophrenia Scale [3], the Positive and Negative Syndrome Scale [4], the Scale for Assessment of Positive Symptoms [5] and the Scale for Assessment of Negative Symptoms [6]. Although the BPRS includes 18 items and the allocation of marks is defined clearly, as all items have the same range of marks (i.e., 1-7), it is not unusual to find that scores for the BPRS differ widely from those for the CGI-SCH.

Ideally, scores from one scale could be mapped directly onto the other, making it possible to compare individuals evaluated with one scale or the other. We decided to investigate this divergence analytically, looking at the clinical weight of respective symptoms (the relative magnitudes of symptoms in schizophrenia) and the issue of scale nonlinearity. In the present study, we investigated whether there was a linear relationship between the scores of the two scales, to observe whether linearity of the BPRS to the CGI-SCH could be influenced by changing point allocation of the BPRS through devising an example of a possible modified BPRS subscale.

The aim of the present study is 4-fold: 1) to investigate the linearity of the BPRS in relation to its items and mark allocation by examining the reasons for the incongruity between BPRS scores and clinicians' impressions of symptom severity in schizophrenic patients; 2) to determine a mathematical expression that represents the relationship between the BPRS and the CGI-SCH more precisely; 3) to seek which symptoms are important from a clinical standpoint; and 4) if possible, to construct an example of a possible modified BPRS subscale that is expected to have improved correlation with the CGI-SCH scores compared with the full BPRS within the limitations of the data obtained in this trial.

## Methods

### Participants

This was a retrospective study of outpatients and inpatients treated at the Tokyo Women's Medical University, Miyazaki Hospital and Depression Prevention Medical Center, Kyoto Jujo Rehabilitation Hospital, Japan, who met the DSM-IV-TR [7] criteria for schizophrenia. A total of 150 patients (74 males, 76 females) with a mean age of 44.5 years (range, 17-83) were included in this study. Fifty patients were suffering their first episode of schizophrenia or attending for initial treatment (Group A) and 100 were randomly selected during either the acute or chronic phase of schizophrenia (Group B). The study involved a retrospective chart review and was approved by the ethics committee of our institution.

### Research design

All patients were evaluated and rated from their medical records using the BPRS and the CGI-SCH during the same session, but at initial consultation for Group A and at a random treatment session for Group B. In this study, we utilized the CGI-SCH as a scale that substituted for the evaluation made by the patients' psychiatrists under the tentative assumption that the CGI-SCH had perfect linearity and that it represented the precise clinical global impression of the treating psychiatrists.

If the linearity of the BPRS to the CGI-SCH was not initially apparent, we aimed to derive a mathematical equation to represent more precisely the relationship between the scales, clarifying which symptoms were important in evaluating schizophrenia and how we could improve the correlation between the BPRS and the CGI-SCH. Two experienced psychiatrists shared their evaluations, and the scores for the BPRS and the CGI-SCH were presented graphically, making it possible to examine whether the two demonstrated a linear relationship. At this stage, we examined the distribution on the scatter plot of the two scales and expressed the relationship in a precise mathematical equation. Next, backward stepwise regression was performed, with the CGI-SCH as a dependent variable and with all 18 items of the BPRS as independent variables. An F-value of less than 2.000 was used to identify variables for removal. In addition, backward stepwise regression with F-value at the same condition was performed using three small sets of variables based on derived scores. These independent variable groups were: positive symptoms (conceptual disorganization, grandiosity, hostility, suspiciousness, hallucinations, and excitement); negative symptoms (emotional withdrawal and blunted affect); and general psychopathological symptoms (somatic concern, anxiety, guilt, tension, bizarre behavior, depressed mood, motor retardation, uncooperativeness, unusual thought content, and disorientation) with reference to three domains of the PANSS. Variables showing a positive association with the CGI-SCH were derived from these three domains, and multivariate regression analysis was performed using the selected items as independent variables and the CGI-SCH as a dependent variable. By convention in stepwise regression, even if p values exceed 0.05, it is permissible to adopt the variables if those symptoms are judged as clinically important, as long as the p values do not exceed 0.20. However, we selected the variables positively associated with the CGI-SCH within the condition that p values were less than 0.05. In the stepwise and multivariate regression analyses, we often obtained variables inversely associated with the CGI-SCH. In this study, we adopted a way to remove them. Furthermore, utilizing the results of multivariate regression, we allocated marks in proportion to the magnitude of the multiple regression coefficient of each variable so that each symptom was allocated different marks proportional to the positive multiple regression coefficient.

With regard to linearity, we then examined the distribution on the scatter plot of the BPRS and the CGI-SCH scores. We obtained the Pearson's r coefficient as an indication of the degree of linearity of the relationship between the two scales, r-squared being one of values used to estimate how much the fit of model shrinks (by observing how r-squared decreased), before and after exclusion of the various items and modification of the mark allocation. Furthermore, we examined whether the selection of specific items and/or changing the distribution of the marks enhanced the correlation of the BPRS with the CGI-SCH and how much the r-squared decreased. On the basis of the results, we constructed an example of a possible modified BPRS subscale, "the modified seven-item BPRS", which would be expected to have a higher correlation with the CGI-SCH within the limitations of the applicability for our data at this stage. We used SPSS for Windows, version 14 [8] for the stepwise regression analysis, Stata Release 10.0 [9] for the multivariate regression analysis, and Microsoft Excel 2003 [10] for plotting the graph.

## Results

**Figure 1:**
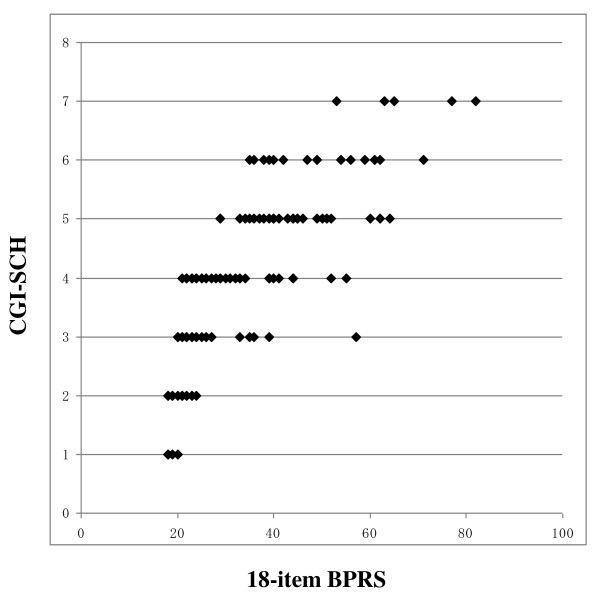
**Scatter plot of the 18-item BPRS total score and the CGI-SCH score**. An upper convexity curve similar to a logarithmic curve was evident, and a linear relationship was not apparent. The range of the 18-item BPRS was 18-126, and that of the CGI-SCH was 1-7.

**Figure 2:**
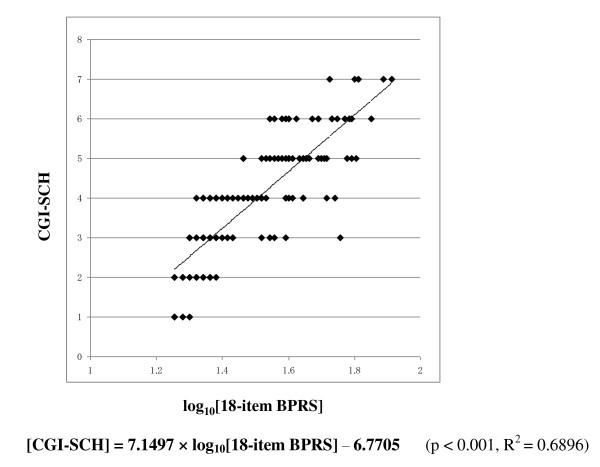
**Scatter plot of the common logarithm of the 18-item BPRS total score and the CGI-SCH score**. After performing a common logarithmic transformation on the 18-item BPRS score, the approximately logarithmic curve was modified to an almost linear distribution and the increase in the common logarithm of the 18-item BPRS total score was almost proportional to the increase in the CGI-SCH score.

**Figure 3:**
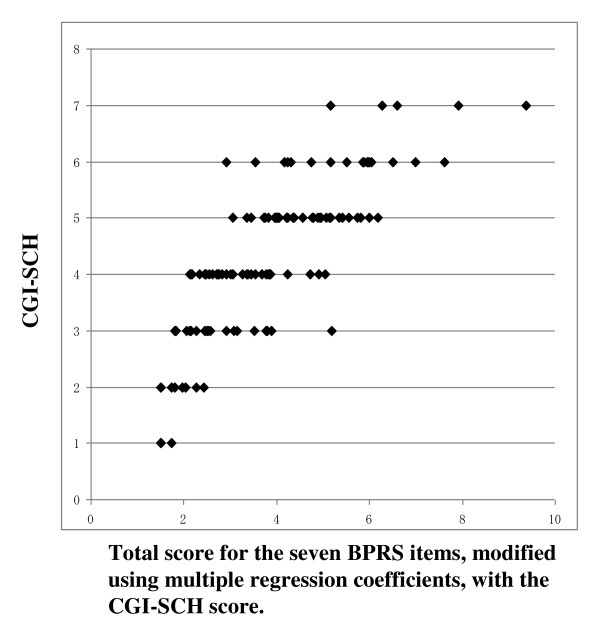
**Scatter plot of the seven-item total score, modified using multiple regression coefficients, and the CGI-SCH score**. The score for each of the seven items was multiplied by the multiple regression coefficient for each respective symptom. The range of the total score for the seven BPRS items modified using multiple regression coefficients was 1.497-10.479, and that for CGI-SCH was 1-7. The sum of the regression coefficients for the seven variables positively associated with the CGI-SCH score was 1.497.

**Figure 4:**
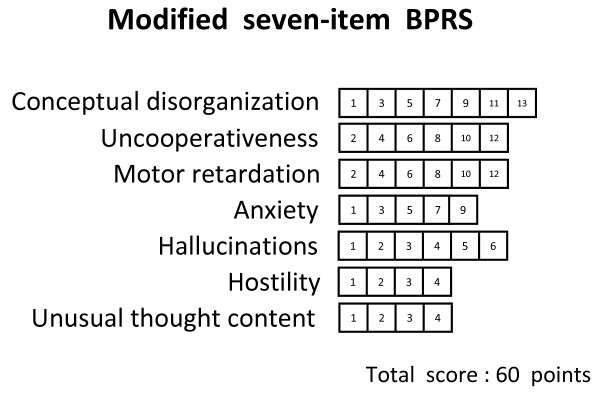
**An example of a possible modified BPRS subscale**. Marks for each item were obtained by multiplying the respective regression coefficient for the seven selected items by 40.

Figure 1 shows the relationship between the 18-item BPRS score and the CGI-SCH score. Although there was a rough correlation, a curve with upper convexity was obtained, and the straight-line relationship that had been thought to exist between the two scales was not apparent. Because the shape of the curve was similar to a logarithmic curve, we performed a logarithmic transformation of the 18-item BPRS total score. The curve was then modified to an almost linear distribution, which was described by the equation [CGI-SCH] = 7.1497 × log_10_[18-item BPRS] - 6.7705 (p < 0.001; Figure 2). Pearson's r coefficient for the relationship between the 18-item BPRS and the CGI-SCH was 0.7926 (p < 0.001) and r-squared (that of multivariate regression using the full item of BPRS) was 0.7560. The results of backward stepwise analysis for correlation are shown in Table 1 (p < 0.001). According to the results of other backward stepwise regressions for variables divided into three groups, eight items were selected (p < 0.001; Table 2). 'Conceptual disorganization' (p < 0.001), 'hostility' (p < 0.001), 'hallucinations' (p < 0.001), 'emotional withdrawal' (p < 0.001), 'anxiety' (p < 0.001), 'motor retardation' (p < 0.001), 'uncooperativeness' (p = 0.004) and 'unusual thought content' (p < 0.001) were significantly correlated with the CGI-SCH. The selection of the above eight variables from the 18-item BPRS gave Pearson's r of 0.8185 and r-squared of 0.7198. Using these eight items as independent variables that were expected to be important for the correlation between the BPRS and the CGI-SCH, multivariate regression analysis was performed (Table 3). After further selection of the seven variables from the above eight variables at the multivariate regression, "the seven-item BPRS" was obtained that comprised these positively associated seven items. Pearson's r for the relationship between "the seven-item BPRS" and the CGI-SCH was 0.8315 and r-squared was 0.7036 (p < 0.001). Furthermore, because we were able to consider the weight of the multiple regression coefficient as the clinical weight, the standard deviation of each variable was assumed to be almost the same, and by allocating marks to each respective item of "the seven-item BPRS" in proportion to the magnitude of the multiple regression coefficient, Pearson's r was increased further to 0.8339 (between "the modified seven-item BPRS" and the CGI-SCH; p < 0.001; Figure 3) and r-squared did not change (0.7036). As a result, the distribution on the scatter plot of the two scales changed from that shown in Figure 1 to that shown in Figure 3, yielding a more linear relationship between "the modified seven-item BPRS" and the CGI-SCH than was the case between the 18-item BPRS and the CGI-SCH. Pearson's r was increased after the series of manipulations, and r-squared decreased by slow degrees, although statistical significance was not apparent for this change. By multiplying each multiple regression coefficient by 40, we composed an example of a possible modified BPRS subscale: "the modified seven-item BPRS" (of tentative meaning, given the limits of our data at this stage) (Figure 4).

**Table 1:** Results of stepwise regression 1.

Variable	Unstandardized β	Standard Error	Standardized β	t	p-Value	95% Confidence Interval
Somatic concern	-0.253525**	0.078032	-0.171	-3.249	0.001	-0.407828 - -0.099222
Anxiety	0.413167^†^	0.082008	0.411	5.038	0.000	0.251002 - 0.575332
Emotional withdrawal	-0.351087**	0.106518	-0.324	-3.296	0.001	-0.561719 - -0.140454
Conceptual disorganization	0.358483^†^	0.069136	0.353	5.185	0.000	0.221771 - 0.495195
Grandiosity	-0.189250*	0.088471	-0.099	-2.139	0.034	-0.364195 - -0.014304
Hostility	0.166995	0.104009	0.140	1.606	0.111	-0.038676 - 0.372666
Suspiciousness	-0.152787	0.091570	-0.164	-1.669	0.097	-0.333861 - 0.028287
Hallucinations	0.162475**	0.057716	0.204	2.815	0.006	0.048346 - 0.276605
Motor retardation	0.258352**	0.090433	0.190	2.857	0.005	0.079527 - 0.437178
Uncooperativeness	0.262802*	0.109879	0.239	2.392	0.018	0.045524 - 0.480081
Unusual thought content	0.147787*	0.073080	0.162	2.022	0.045	0.003277 - 0.292296
Blunted affect	0.139060	0.079696	0.095	1.745	0.083	-0.018534 - 0.296652

Constant	1.138607	0.256029		4.447	0.000	0.632328 - 1.644886

*p < 0.05, **p < 0.01, ^†^p < 0.001

Results of backward stepwise regression using the full set of variables of the BPRS. Eight items of the BPRS as independent variables were positively associated with the CGI-SCH score as a dependent variable, and four items were inversely associated with the CGI-SCH score. F-value < 2.000 as a criterion for removal (p < 0.001). R-squared was 0.7524 (p value of analysis of variance was less than 0.001), unstandardized β, standardized β, and the p value are shown.

Data for schizophrenic patients (n = 150)

**Table 2:** Results of stepwise regression 2.

Positive symptoms (conceptual disorganization, grandiosity, hostility, suspiciousness, hallucinations and excitement)						
**Variable**	**Unstandardized β**	**Standard Error**	**Standardized β**	**t**	**p-Value**	**95% Confidence Interval**

Conceptual disorganization	0.419673^†^	0.062355	0.414	6.730	0.000	0.296437 - 0.542909
Hostility	0.247835^†^	0.066360	0.208	3.735	0.000	0.116685 - 0.378984
Hallucinations	0.288605^†^	0.047700	0.363	6.050	0.000	0.194333 - 0.382877

Constant	1.586381	0.163776		9.686	0.000	1.262703 - 1.910059

**Negative symptoms (emotional withdrawal and blunted affect)**						

Variable	Unstandardized β	Standard Error	Standardized β	t	p-Value	95% Confidence Interval

Emotional withdrawal	0.668840^†^	0.069960	0.618	9.560	0.000	0.530592 - 0.807089

Constant	2.624571	0.167903		15.631	0.000	2.292775 - 2.956368

**General psychopathological symptoms (somatic concern, anxiety, guilt, tension, bizarre behavior, depressed mood, motor retardation, uncooperativeness, unusual thought content and disorientation)**						

Variable	Unstandardized β	Standard Error	Standardized β	t	p-Value	95% Confidence Interval

Somatic concern	-0.220833**	0.082965	-0.149	-2.662	0.009	-0.384830 - -0.056837
Anxiety	0.251795^†^	0.066748	0.250	3.772	0.000	0.119856 - 0.383734
Motor retardation	0.303597^†^	0.078253	0.224	3.880	0.000	0.148916 - 0.458278
Uncooperativeness	0.223711**	0.076051	0.204	2.942	0.004	0.073381 - 0.374041
Unusual thought content	0.350995^†^	0.062647	0.386	5.603	0.000	0.227160 - 0.474829
Disorientation	0.244946	0.168480	0.076	1.454	0.148	-0.088087 - 0.577979

Constant	1.430263	0.234066		6.111	0.000	0.967587 - 1.892939

^†^p < 0.001 Data for schizophrenic patients (n = 150)

^†^p < 0.001 Data for schizophrenic patients (n = 150)

*p < 0.05, **p < 0.01, ^†^p < 0.001 Data for schizophrenic patients (n = 150)

Results of backward stepwise regression using small sets of variables based on the three domains of the PANSS (positive symptoms, negative symptoms and general psychopathological symptoms). Within positive symptoms, 'conceptual disorganization', 'hostility' and 'hallucinations' were significantly and positively associated with the CGI-SCH score (p < 0.001); within negative symptoms: 'emotional withdrawal' (p < 0.001); within general psychopathological symptoms: 'anxiety', 'motor retardation', 'uncooperativeness', and 'unusual thought content' were significantly and positively associated with the SCH-SCH score (p < 0.005), and 'somatic concern' was inversely associated with the CGI-SCH score (p < 0.01). F-value < 2.000 as a criterion for removal, multiple regression coefficient and the p value are shown. R-squared was 0.6661 (positive symptoms), 0.3818 (negative symptoms) and 0.6716 (general psychopathological symptoms); all p values of respective analysis of variance were less than 0.001.

**Table 3:** Results of multivariate regression.

Variable	Multiple Regression Coefficient	Standard Error	t	p-Value	95% Confidence Interval
Conceptual disorganization	0.325896^†^	0.071154	4.580	0.000	0.185228 - 0.466563
Hostility	0.103284	0.080584	1.282	0.202	-0.056024 - 0.262592
Hallucinations	0.139101*	0.059103	2.354	0.020	0.022258 - 0.255944
Emotional withdrawal	-0.312958**	0.109653	-2.854	0.005	-0.529734 - -0.096182
Anxiety	0.235261**	0.068236	3.448	0.001	0.100363 - 0.370158
Motor retardation	0.297271**	0.086420	3.440	0.001	0.126424 - 0.468118
Uncooperativeness	0.300436**	0.111556	2.693	0.008	0.079897 - 0.520976
Unusual thought content	0.095707	0.072111	1.327	0.187	-0.046852 - 0.238266

Constant	1.093726	0.193951	5.639	0.000	0.710298 - 1.477155

*p < 0.05, **p < 0.01, ^†^p < 0.001

Relative weights of the variables (which were selected as being positively and significantly associated with the CGI-SCH score with the p value < 0.05 in Table 2) are presented as a set of magnitudes of multiple regression coefficients (p < 0.001). R-squared was 0.7198 (the subset of eight items resulted from stepwise regression using three small sets) and p value of analysis of variance was less than 0.001.

Data for schizophrenic patients (n = 150)

## Discussion

The BPRS is one of the most frequently used instruments for evaluating the psychopathology of patients with schizophrenia. Although its psychometric properties in terms of reliability, validity and sensitivity have been extensively examined [11], patients are examined by clinicians with different observer ratings using different criteria. On the other hand, assessment with the CGI-SCH is based on a score of 1-7, making it simple and relevant. The CGI-SCH may be as sensitive as the BPRS in detecting efficacy differences between antipsychotic drugs [12], but it is necessary that treatment response be interpreted in the context of patient characteristics [13]. However, patients with different characteristics but with similar scores are often treated similarly in clinical trials. Therefore, training is required for performing a standardized evaluation [14]. Other user-friendly assessments include the Revised Global Outcome Assessment of Life in Schizophrenia (Revised GOALS) [15], the Investigator's Assessment Questionnaire (IAQ) [16] and the Targeted Inventory on Problems in Schizophrenia (TIP-Sz) [17], although they have some limitations in terms of methodology. We also believe that other important aspects of illness management should be supplemented with appropriate subjective scales as necessary [18]. Nonetheless, there is no consensus among clinicians regarding the most suitable scale. To address this perplexing issue, more advanced investigations are necessary to devise rating scales using some form of statistical method.

Leaving aside the debate over whether psychopathological severity or state can be expressed in evaluation scales such as the BPRS or the CGI-SCH and accepting the need and utility of such instruments, we focused here on improving the BPRS scale. We examined whether the more detailed assessment afforded by its items and individual point allocations could be made proportional to the simpler and more global CGI-SCH scale.

Many previous attempts have been made to evaluate the adequacy of the BPRS from the viewpoint of which items should be selected because of their relevance. However, no study has approached this issue by addressing how the degree of linearity of the BPRS can be changed by modifying not only its constituent items but also their weighting, using stepwise regression and multivariate regression analysis. In the present study, we first examined whether the BPRS and the CGI-SCH showed a mutual linear relationship. By plotting the BPRS scores and the CGI-SCH scores in the form of a graph, we compared their respective distributions. The scatter plot of the 18-item BPRS and the CGI-SCH yielded a curve with upper convexity, thus demonstrating that the relationship between the two scales was not linear (see Figure 1). Because the shape of the curve had upper convexity similar to a logarithmic curve, we performed a common logarithmic transformation on the 18-item BPRS score. Then, we were able to obtain a possibly more precise equation as a logarithmic form shown in Figure 2. From this result, we presumed that there was a possibility that an increase in the logarithm of the total score for all symptoms might be roughly proportional to the global increase in symptom severity observed clinically in schizophrenic patients. We recognize, however, that this model has applicability to only this trial at this stage. We inferred that the logarithmic relationship between the single score scale, the CGI-SCH, and the plural score scale, the BPRS, might be an important tool in our determination of the severity of illness. We then investigated whether modifying the constituent items and/or allocation of marks could affect the linearity of the BPRS, at least, within this trial itself, looking at the correlation of the BPRS with the CGI-SCH in terms of Pearson's r and r-squared, which express one of the degrees of the fit between the two scales. To evaluate the clinical severity of schizophrenia, we substituted the CGI-SCH score for the clinical impression.

Partly because the values of Pearson's r were slightly higher between "the seven-item BPRS" (constructed by selection of specific items) and the CGI-SCH than between the 18-item BPRS and the CGI-SCH without considerable decreases of r-squared, the shape of the scatter plot between the two scales became more linear than that before the selection. We inferred that there was a possibility that the selection of these items from 18 items is related to the linearity of the BPRS. The clinical weights might be related to the heightened values of Pearson's r between "the modified seven-item BPRS" (constructed by changing the allocation of marks) and the CGI-SCH, as the shape of the scatter plot between the two scales became more linear than before. We presumed that there was a possibility that the weighting was also associated with the linearity of the BPRS.

Furthermore, by assigning different weights to each item proportional to the respective regression coefficients, we were able to compose a possible modified BPRS subscale, "the modified seven-item BPRS", by assuming that the magnitude of each regression coefficient represented the respective clinical weight of each item. This scale is only an example of a possible modified BPRS subscale that we are able to assume within our data, and the number of items decreased from 18 to 7.

Historically, a widely used algorithm employing a stepwise method was first proposed by Efroymson [19], and supplementary articles were later reported by Hocking [20] and others. In the field of psychiatry, stepwise methods have been used for predicting the quality of life of schizophrenic patients by reference to schizophrenia symptoms [21], for estimating predictive values of neurocognition in schizophrenic patients [22], and for estimation of the relationship between executive functions and positive symptoms in schizophrenia [23].

However, some problems with stepwise and multivariate regression analysis have been reported. To compare the relative magnitudes of variables, the partial regression coefficients are often normalized using their respective standard deviations. However, the predictor variable is at least partially redundant with other predictors and the regression coefficient is influenced by the range of the predictor variable [24]. In addition, the relative importance of predictor variables is a tenuous concept, and comparison of the importance of predictors is not always the best approach in multiple regression. As the individual items of the BPRS had the same range of marks (1-7), we considered that there would not be crucial differences in the sizes of standard deviations for predictor variables in this study. With this assumption, we considered that, for practical purposes, the magnitudes of the standardized and unstandardized β might be regarded as almost equivalent. For these reasons, we utilized the magnitude of the unstandardized β (multiple regression coefficient) to modify the distribution of marks of the BPRS and to design a tentative BPRS subscale. If supplemented with this adjustment, the scatter plot representing the relationship between "the modified seven-item BPRS" and the CGI-SCH showed a distribution proportional to the scatter plot connecting the score of "the seven-item BPRS" multiplied by the unstandardized β (multiple regression coefficient) for each item and the score of the CGI-SCH. This is because both have almost the same significance on the graph. Additional improvements in fit may be possible.

The limitations of the present study should be noted. The first was the use of the CGI-SCH as a scale that substituted for the evaluation made by the patients' psychiatrists. There is no evidence that the CGI-SCH has perfect linearity and this was merely an assumption to allow modification of the BPRS under a determinate condition. For the CGI-SCH, only a certain degree of reliability has been reported [3,12,13]. Nonetheless, we thought that this kind of simplification was unavoidable and the trade-off necessary, even if this assumption would sacrifice rigor to some extent in exchange for examining the degree of an abstract value such as 'linearity.'

Second, there is no evidence supporting the assumption that the BPRS score and the CGI-SCH score obtained retrospectively by coding of the symptoms reported in the clinical chart would be comparable to the data obtained from trained BPRS raters monitored for inter-rater reliability and performing standardized interviews to probe for presence and severity of a complete list of symptoms. The quality of the clinical chart is notoriously variable, so there may exist errors and distortions from missing symptoms and falsely rating symptoms as absent when reviewing a chart, because of the failure of the clinician to mention them in the chart, which would have been detected in a structured, face-to-face interview. The 0.7926 value of Pearson's r might be to some extent considered high. However, we presume that this was because the study was retrospective. Therefore, some items of the BPRS might not have been marked, thus minimizing the distribution of the BPRS score. In effect, the results of this paper may be applicable only within our own data at this stage (including the derived stepwise regression, multivariate regression and, particularly, "the modified seven-item BPRS") and there is no guarantee that the results would be comparable to prospective research. From this standpoint, our report might be regarded as one of these experimental case studies. At any rate, prospectively randomized trials are needed in future studies.

Third, through the manipulations employed here, the degree of change in Pearson's r was rather ambiguous. The increases appear slight (as a total, from 0.7926 to 0.8339; particularly, in the final manipulation, from 0.8315 to 0.8339). Moreover, it is considerably uncertain whether statistical significance exists. From another viewpoint, despite the fact that the scale was simplified, and in particular, the number of items decreased, the degree of correlation (Pearson's r) stayed at the same level or increased just slightly. Although this might indicate that a simplified subscale might be valuable in comparison with the full scale, and it might be useful to clinicians for shortening time of rating, the reproducibility of items and point allocation is quite uncertain in this model. We believe that a study of this theme in the future is desirable.

Fourth, as for r-squared, in general, the more variables we exclude from the model, the more r-squared tends to decrease. The selection of the subset for which the decrease of r-squared is smallest is preferred so that the loss of model fit would be minimal. The r-squared of our data ranged from about 0.70-0.75. The size of these numbers is not low, but they may not be sufficiently high even with the moderate degree of decrease. For example, 0.7560 for the full item BPRS; 0.7524 for the results of stepwise regression using the full items of BPRS; 0.7198 for the selected eight items from stepwise regression using three small sets; and 0.7036 for the selected seven items from multivariate regression (all p values of respective analysis of variance were less than 0.001). This means that the selection of items might have caused shrinkage of the model.

Fifth, the selection of items and modification of point allocation may have contributed some artifacts of multicollinearity. There are likely to be intercorrelations among the data. In this study, variables that were inversely correlated with the CGI-SCH score, indicating that the more severe the BPRS item, the lower the CGI-SCH score (a phenomenon which was a departure from the clinicians' experiences), were simply excluded from the model in an ad hoc procedure. This ignored the fact that the selection of the other predictors from among the list of candidates depended on the presence of the excluded variable. Additional unknown and complicated factors are predicted to exist as well, for example, that both inpatients and outpatients were evaluated by the CGI-SCH, and that the results might have been negatively influenced by differences in cognitive ability [25]. The treatment of negative coefficients is a crucial weakness of our model.

Sixth, above all, the results are not likely to be reproducible. If we performed the same procedure on new data, it is very likely that different symptoms would be selected, and different point allocations would probably be assigned to individual items. We infer that a possible way to remedy this problem, even if partially, might be to perform a number of prospective trials in line with our method, and then summarize and calculate an average on items and point allocation. If these scales are composed as a summary, they might be less problematic than that of our trials. However, even in such scales, there would still be no assurance that they would have a greater degree of reproducibility. Therefore, the extent to which the results of this paper could be applicable may be quite limited: at the extreme, only within our present data. For this reason, future studies are necessary.

The true aim of this manipulation was not always to determine the best subset and/or point allocation, but to consider a specific example of a possible modified scale. Therefore, "the modified seven-item BPRS" is merely a tentative idea at this stage, to propose a new viewpoint of the importance of point allocation in the BPRS. Needless to say, the present study has many limitations, and is thus only a first step from which further studies may learn. We believe that improving evaluation scales to make them more linear could minimize distortions in evaluation for severity of illness, including over- and under-diagnosis and estimations for efficiency and effect in clinical research. We anticipate that our present results will serve as a useful reference for clinicians attempting to devise an evaluation scale, and that further research will focus on the optimal number of items, the fittest items for selection, and the allocation of marks in rigorous methodology to maximize the linearity of the BPRS.

## Conclusions

Within the limits of our data, although there was a rough correlation, the linear relationship that had been thought to exist between the 18-item BPRS and the CGI-SCH was not apparent. Also, a roughly logarithmic relationship was assumed between the two scales. In addition, not only specific items but also their weightings were considered to be important in the realization of a linear relationship between the BPRS and the CGI-SCH and in the further improvement of the BPRS as a diagnostic scale.

## Competing interests

The authors declare that they have no competing interests.

## Authors' contributions

JS performed the evaluation of the patients and the statistical analysis, and wrote the manuscript. SM also performed the evaluation of the patients and revised the manuscript. JI was responsible for checking the methodology of the study and evaluating the results of the statistical analysis. In addition, all authors read and approved the final version of the manuscript.

## Pre-publication history

The pre-publication history for this paper can be accessed here:

http://www.biomedcentral.com/1471-244X/10/105/prepub
